# Polypiform Concretions in the Cavities of the Heart, with a Case

**Published:** 1858-03

**Authors:** P. A. Allaire

**Affiliations:** Aurora, Illinois


### ARTICLE III.

### POLYPIFORM CONCRETIONS IN THE CAVITIES OF THE HEART, WITH A CASE.

BY P. A. ALLAIRE, M. D., AURORA, ILLINOIS.

The concretions found in the heart, commonly classed as polypi of that organ, have been usually divided into three kinds, viz: 1, Concretions of fibrine; 2, Concretions of lymph; and, 3, Concretions composed of lymph and fibrine. If a fourth variety is made, it must be by including that coagulum of blood which is formed either post-mortem or during the last moments of life, and which is occasionally found coated with fibrine; but such are truly coagula, they illustrate the mode of death, and are not evidence of pre-existing disease.

1. *Concretions of fibrine, or unorganized,* are doubtless the most frequent, as they occur in those cases in which an impediment has existed to the circulation, as in various valvular and other non-inflammatory heart diseases, asthma, anæmia, and cases of sudden exhaustion as by hemorrhage. This concretion is of a straw color, semi-elastic, and is adherent, but not intimately to the columnæ carnæ and tendinous cords of the valves. *The fibrine in these concretions is mechanically separated from the blood,* a coagulum having been first formed in the heart. The size of these polypi is usually sufficient to greatly embarrass the circulation, yet life may continue a long time after such an occurrence; or if the concretion is not large, it may be, and doubtless often is, removed from the heart, though often the obstruction is sufficient to cause speedy death. I have known one case of this disease caused by post-partum hemorrhage, where, after two years' suffering, the recovery was complete. The symptoms were—a bellows murmur, feeble pulse, shortness of breath, and palpitation on exertion.

2. *Concretions of lymph or organized.* These are quite different in origin from the fibrinous kind, being a result of inflammation of the endocardium, a true deposit of coagulable lymph. They readily become organized, and are more intimately adherent to the columnæ carnæ, etc., than the first mentioned variety. Their size is usually not so great, hence they are not apt to be so speedily fatal. This form of concretion is supposed to be the origin of the warty and globular vegetations described by Laennec, and hence the cause of many of the chronic valvular and other obstructive diseases of the heart, which result in hypertrophy and dilatation of that organ. These polypi are of a lighter color than the first, more dense in structure, and organized, but unless seen early after their formation, no red vessels can be detected. The case about to be described was of this variety, and is remarkable for the large size of the concretions and the duration of the disease.

3. *Concretions of lymph and fibrine, or partially organized.*

The two former varieties are combined in this, the nucleus being lymph. The cause is the same as in the second variety; in reality, it is the second variety suddenly increased in severity, from the size of the polypus being augmented by some new embarrassment to the circulation, causing a deposit of fibrine on the pre-existing concretion, thus increasing the size often to that of the first kind.

None of the forms of polypi of the heart give rise to certain symptoms; diagnosis is, consequently, difficult. They may be inferred from the irregular, confused pulsations of the heart, the dyspnœa, the diminished supply of arterial blood, and, generally, the presence of more or less venous congestion. The symptoms are often irregular, or even ceasing for a time, owing to change in the position of the mass. No means are known by which these concretions can be removed or dissolved; the treatment becomes, therefore, merely palliative, as does, also, the treatment for the secondary affections of the liver, lungs, etc., resulting from the venous congestion of those organs in this and nearly all other cardiac affections of a chronic character.

Case.—Helen P. was first seen by me as a patient, December 29th, 1857. She is now eleven years old, of healthy parents, and well developed; formerly an active child; she is now very quiet, moving about with great care, frequently complaining of a general feeling of uneasiness in the left side of the chest. This sensation commenced about a year since, and has gradually increased. Five years ago, she had an attack of disease called then lung fever; there was active fever, cough, and pain in the left side. "From this she never seemed entirely to recover." There was constant dyspnœa and palpitation, increased on making active exertion; also, frequent attacks called asthma, attended with fever, and terminating in free mucous expectorations. These have been usually of short duration, leaving only the usual dyspnœa and palpitation. Still, the child grew finely, and was supposed by physician and parents to be only asthmatic. Within the past year she has had fewer asthmatic attacks, and has been more quiet and more confined to the house; the last attack, six months since, she recovered from as usual. Her indisposition to make exertion has greatly increased within the last few weeks, until now, when she scarcely makes any attempt to move about.

*Present condition.*—No emaciation, face pale and slightly puffy, lips a little blue, slight œdema of feet and hands, urine scanty, bowels regular and stools healthy, slight cough, and mucous expectoration; respiration, thirty per minute. The action of the heart elevates the ribs at each pulsation, and is 100 to 105 in the minute. *No pulsation can be felt at either wrist*, and it is quite feeble in the carotids. The jugulars are not distended. On placing the hand over the heart, there is a peculiar, indistinct, undulating motion felt, and it is even difficult to count thus the heart's contractions.

The extent of cardiac dulness is doubled. On applying the ear to the region of the heart, no natural sound can be heard, but there is an imperfect gurgling and some approach to a bellows murmur, but nothing distinct. The pulsations can be heard in every part of the chest. The respiration is peurile in both lungs, with slight mucous rhonchus. No examination was made at this time of the abdominal organs. On informing the parents that the case was one of heart disease, they were much surprised, having hitherto regarded the child as only asthmatic. No treatment was at this time instituted, and two days after, viz: December 31st, in the evening, I was requested to see Helen P. again. She had had about noon a slight chill, soon followed by high fever; dyspnœa and palpitation increased; cough dry, with pain in left side; headache, no costiveness, tongue clean. No pulse can be felt at the wrist; pulsations of heart 140, and respiration 43 per minute; slight crepitant rale in left lung; right lung as usual. On inspection, the chest seems more rounded than in health, and the intercostal spaces widened; percussion gives a very clear sound, except over the heart; enlargement of liver is distinct, and upper part of abdomen is unusually full. Regarding the present attack as one of acute bronchitis, the following prescription was made:

| | | | |
|---|---|---|---|
| ℞ Ant. et potass. tart., | gr. ij | Tinct. opii, | ʒj |
| Pulv. ipecac., | gr. x | Water, | ℥ij |

Mix, and give every two hours all the patient will bear without vomiting. Hot fomentations to be wrapped around the chest.

*January 1st.*—Respiration and pulsation of heart reduced in frequency; skin moist; slight expectoration; has had some sleep; countenance less anxious; continue same treatment.

*January 2d.*—Improved in every respect; continue same.

*January 3d.*—No fever or pain; much cough and gastric irritation; tongue covered with yellowish coat; frequent vomiting; discontinue antim. and ipecac., and give calomel gr. ss. and Dover's powder gr. iij. every four hours; stimulating liniment to chest and abdomen.

*January 4th.* — Gastric irritation slight; rests well; no appetite; cough less; pulse 100, cannot be felt at wrist; cannot take the recumbent position to-day; evidence of a little fluid in abdomen; feet more œdematous. ℞ Continue cal. and Dover's powd., and give cinchonia gr. j. in a little wine every four hours.

*January 5th.*—Much the same; gave a mild purgative, and continued the same remedies.

*January 6th.*— Purgative acted mildly; seems improved; can lie down, and takes nourishment better; still vomits occasionally; continue same treatment.

*January 7th, morning.*—Is more comfortable; strength improved; rested well last night; vomits less, and appetite improved; coughs but little; respiration as easy and comfortable as at any time for some months past; distinguished, for a few moments, a very feeble pulsation at the wrist; discontinue cal. and Dover's powd. and give

℞ Cinchonia, gr. v.
Tinct. valerian.
Spt. æther. nit., aa. ℥ss.

Mix; give half a teaspoonful every three hours in water, and continue liniment to chest. Patient remained without apparent change until 2 o'clock P. M., when she rose up suddenly in bed and fell back—dying in a few moments, without apparent pain or convulsion.

*Autopsy, forty hours after death.* — Pericardium healthy. Heart more than twice as large as the fist of the individual, but its proportions were well maintained throughout. The valves were all healthy. In the left ventricle, and attached firmly to the columnæ carnæ and chordæ tendina, was a dense light colored and evidently organized polypiform concretion, which extended into the aorta about one inch, and with a small coagula attached, quite filled it. This concretion would occupy nearly the bulk of a fluid ounce, was about two inches in length, half that measure in width, and one-half inch thick. The sides were irregular, as was also their thickness. Its only attachment was that above mentioned. In the right ventricle was found a concretion similarly but less firmly attached, which extended into and nearly filled the right auricle. This formation was a little larger than the other, less dense, of a yellowish hue, in parts reddish, and here and there striated as if by the rudiments of small vessels. The left ventricle was filled with fluid blood and coagula; the right ventricle was empty. The lungs were free from adhesions, and voluminous; surface a little uneven, and they did not collapse on opening the chest; on being cut into, air escaped with frothy fluid. The air vesicles were enlarged. I regret that the bronchii and their mucous lining were not examined. The liver was at least double the size of the healthy organ, rather more dense in structure than usual; otherwise natural. Gall bladder full and healthy. Spleen natural. Kidneys indurated, lobulated, and slightly diminished in size. On making a section of these organs, the structure seemed natural, except a general shrunken appearance; their fibrous coat could be stripped off with ease, and then the surface of the cortical substance beneath was found studded with firm grayish granules, which were gradually lost in the indurated tissues beneath. No other organs were examined. A small quantity of straw-colored fluid was found in the abdominal cavity. No test was made for albuminous urine.

